# Supplementary material for: JMJD3 regulates the M2-like macrophage polarization and promotes the growth of breast cancer cells via STAT6/IRF4 axis
Source: PLoS One. 2026 Apr 9;21(4):e0341313. doi: 10.1371/journal.pone.0341313 (PMC13065056; doi:10.1371/journal.pone.0341313)
Supplement: S1 Table — (DOCX) [file pone.0341313.s001.docx]

**S1 Table. The relationship between the clinicopathological characteristics and M2 macrophage score in BRCA.**

| **Characteristics** | **Macrophages M2 Score** | | | | ***p* Value** |
| --- | --- | --- | --- | --- | --- |
|  | **High** | | **Low** | |  |
|  | **Total (548)** | **Percentages (%)** | **Total (547)** | **Percentages (%)** |  |
| **Age, n (%)** |  |  |  |  | **<0.001** |
| **<=60** | 241 | 44.7 | 337 | 62.4 |  |
| **>60** | 298 | 55.3 | 203 | 37.6 |  |
| **Pathologic stage, n (%)** | |  |  |  | 0.053 |
| **Stage I** | 93 | 17.5 | 89 | 16.5 |  |
| **Stage II** | 289 | 54.4 | 330 | 61.2 |  |
| **Stage III or IV** | 149 | 28.1 | 120 | 22.3 |  |
| **T stage, n (%)** |  |  |  |  | 0.084 |
| **T1** | 140 | 25.7 | 140 | 25.6 |  |
| **T2** | 303 | 55.6 | 330 | 60.4 |  |
| **T3 or T4** | 102 | 18.7 | 76 | 13.9 |  |
| **M stage, n (%)** |  |  |  |  | 0.782 |
| **M0** | 448 | 97.4 | 462 | 97.9 |  |
| **M1** | 12 | 2.6 | 10 | 2.1 |  |
| **N stage, n (%)** |  |  |  |  | 0.086 |
| **N0** | 249 | 46.5 | 267 | 49.6 |  |
| **N1** | 175 | 32.6 | 186 | 34.6 |  |
| **N2** | 73 | 13.6 | 47 | 8.7 |  |
| **N3** | 39 | 7.3 | 38 | 7.1 |  |
| **Tumor status, n (%)** | |  |  |  | 0.451 |
| **Tumor free** | 425 | 89.5 | 451 | 91.1 |  |
| **With tumor** | 50 | 10.5 | 44 | 8.9 |  |
| **ER, n (%)** |  |  |  |  | **<0.001** |
| **Negative** | 82 | 15.7 | 155 | 29.6 |  |
| **Positive** | 439 | 84.3 | 368 | 70.4 |  |
| **PR, n (%)** |  |  |  |  | **0.002** |
| **Negative** | 147 | 28.3 | 196 | 37.5 |  |
| **Positive** | 372 | 71.7 | 326 | 62.5 |  |
| **Her2, n (%)** |  |  |  |  | 0.119 |
| **Equivocal** | 93 | 20.1 | 86 | 19.5 |  |
| **Neagtive** | 274 | 59.3 | 287 | 64.9 |  |
| **Positive** | 95 | 20.6 | 69 | 15.6 |  |
