# Supplementary figures and images for: JMJD3 regulates the M2-like macrophage polarization and promotes the growth of breast cancer cells via STAT6/IRF4 axis
Source: PLoS One. 2026 Apr 9;21(4):e0341313. doi: 10.1371/journal.pone.0341313 (PMC13065056; doi:10.1371/journal.pone.0341313)

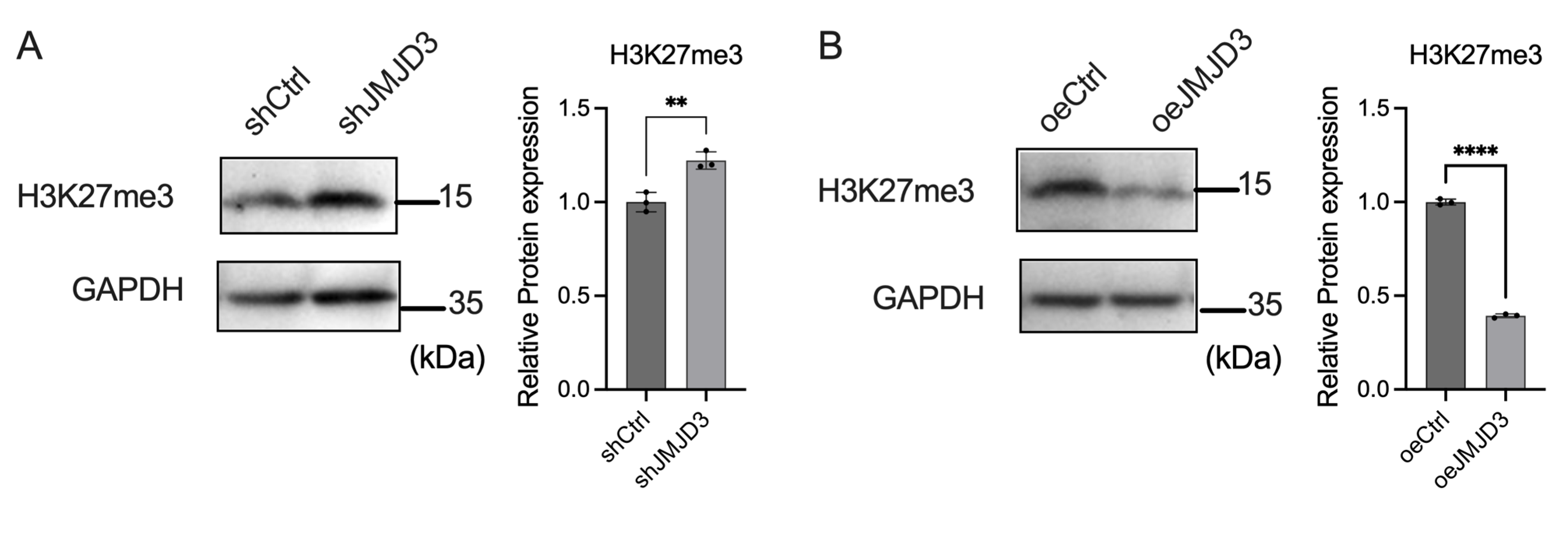

Supplement: S1 Fig — Western blot analysis showing that JMJD3 knockdown elevated H3K27me3 accumulation (A), whereas JMJD3 overexpression reduced H3K27me3 (B) levels in THP-1 cells. GAPDH was used as a loading control. Significance levels: **, p < 0.01, ****, p < 0.001. (TIF) [file pone.0341313.s002.tif]

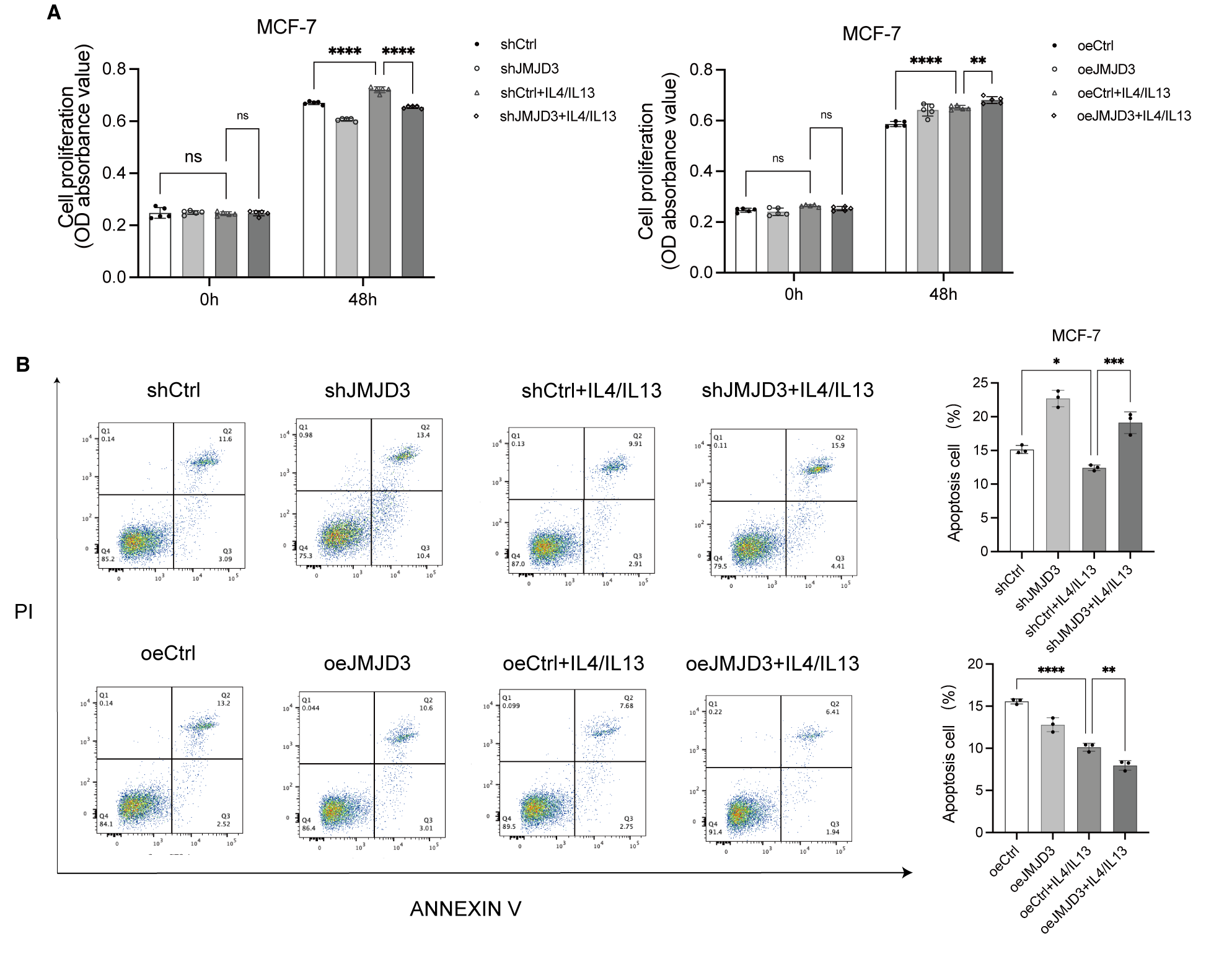

Supplement: S2 Fig — MCF-7 breast cancer cells were cultured with different types of macrophage-conditioned media for 48 hours. A. Proliferation of MCF-7 breast cancer cells assessed using the CCK8 assay. D. Apoptosis rates of MCF-7 cells measured by flow cytometer. Error bars represent mean ± SD. Significance levels: * p < 0.05, ** p < 0.01, ***p < 0.001, ****p < 0.0001. (TIF) [file pone.0341313.s003.tif]
