# Supplementary material for: JMJD3 regulates the M2-like macrophage polarization and promotes the growth of breast cancer cells via STAT6/IRF4 axis
Source: PLoS One. 2026 Apr 9;21(4):e0341313. doi: 10.1371/journal.pone.0341313 (PMC13065056; doi:10.1371/journal.pone.0341313)
Supplement: S1 File — (PDF) [file pone.0341313.s004.pdf]

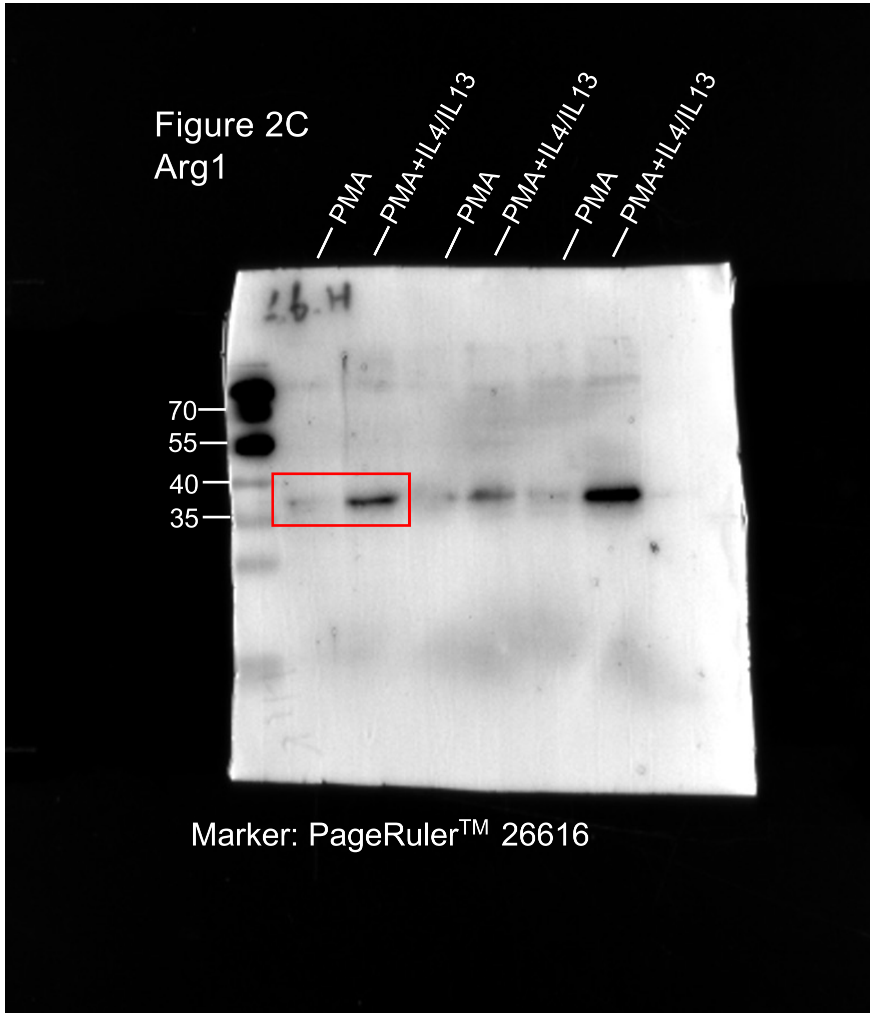
Figure 2C
Arg1
PMA
PMA+IL4/IL13
PMA
PMA+IL4/IL13
PMA
PMA+IL4/IL13
70
55
40
35
Marker: PageRulerTM 26616

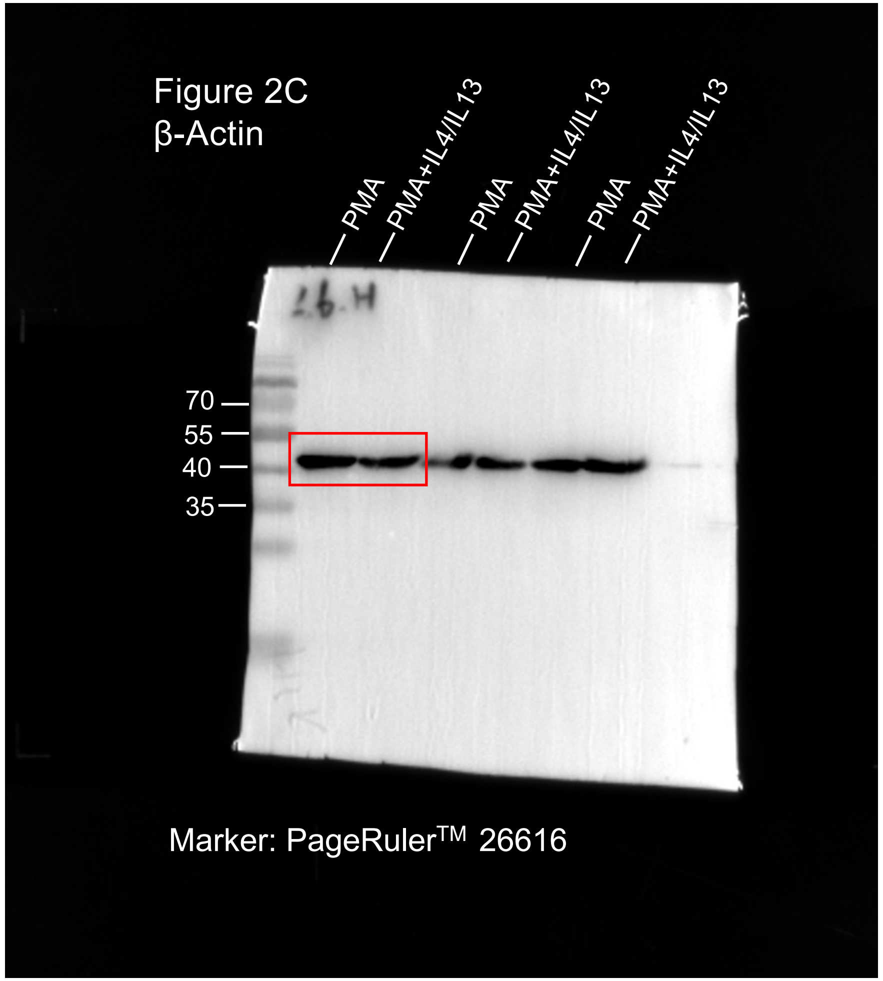
Figure 2C
β-Actin
PMA
PMA+IL4/IL13
PMA
PMA+IL4/IL13
PMA
PMA+IL4/IL13
70
55
40
35
Marker: PageRulerTM 26616

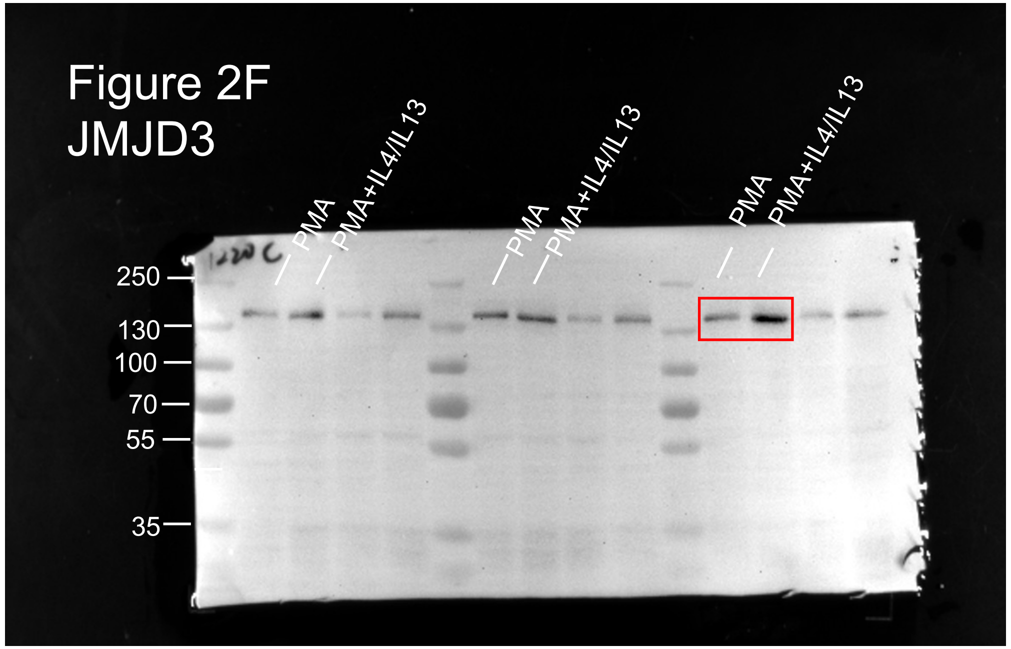
Figure 2F
JMJD3
PMA
PMA+IL4/IL13
PMA
PMA+IL4/IL13
PMA
PMA+IL4/IL13
250
130
100
70
55
35

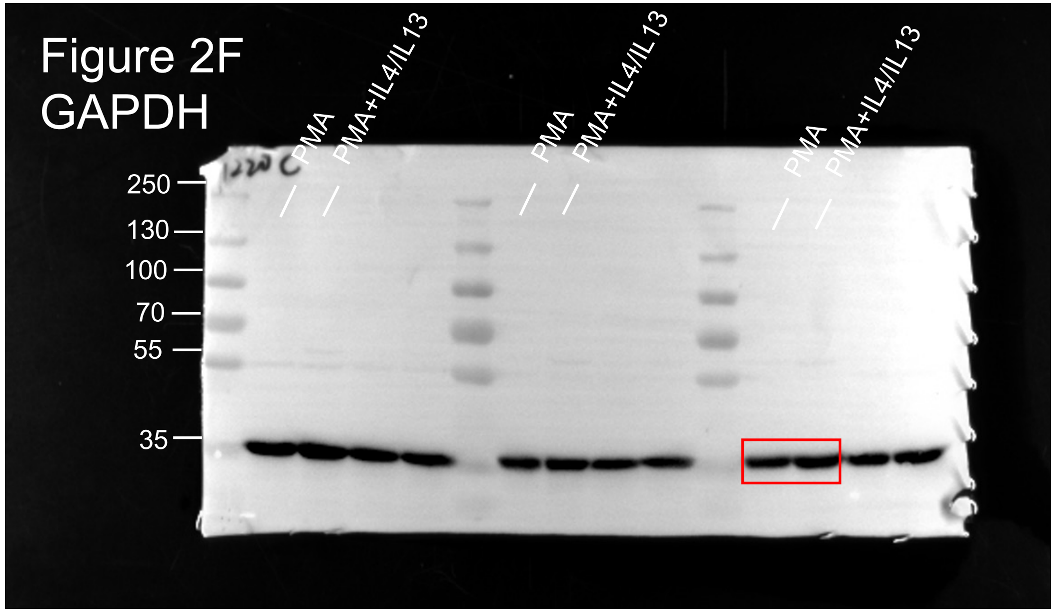
Figure 2F
GAPDH
PMA
PMA+IL4/IL13
PMA
PMA+IL4/IL13
PMA
PMA+IL4/IL13
250
130
100
70
55
35

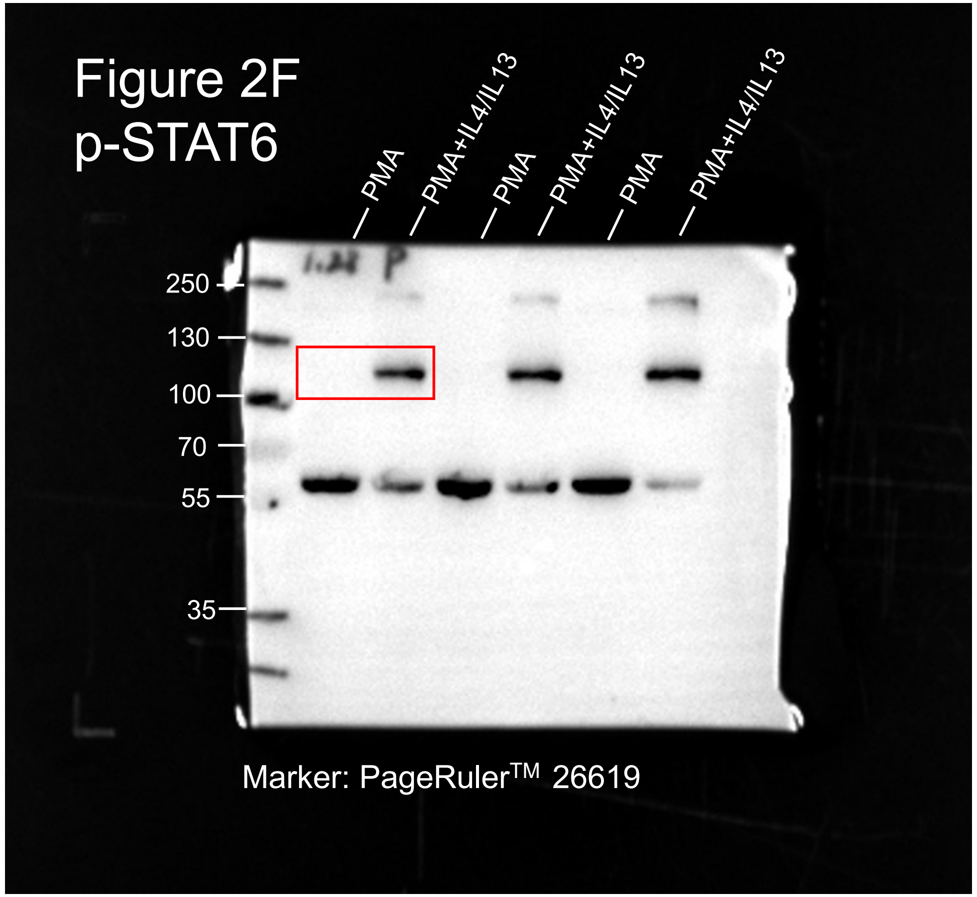
Figure 2F
p-STAT6
PMA
PMA+IL4/IL13
PMA
PMA+IL4/IL13
PMA
PMA+IL4/IL13
250
130
100
70
55
35
Marker: PageRuler™ 26619

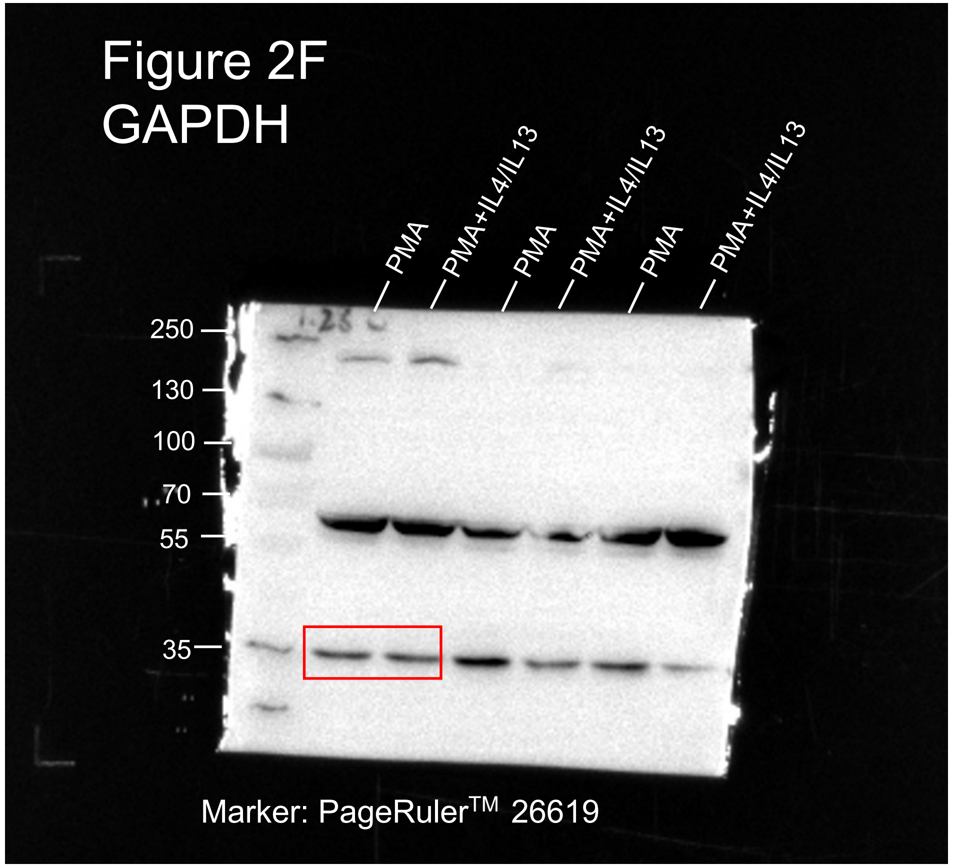
Figure 2F
GAPDH
PMA
PMA+IL4/IL13
PMA
PMA+IL4/IL13
PMA
PMA+IL4/IL13
250
130
100
70
55
35
Marker: PageRuler™ 26619

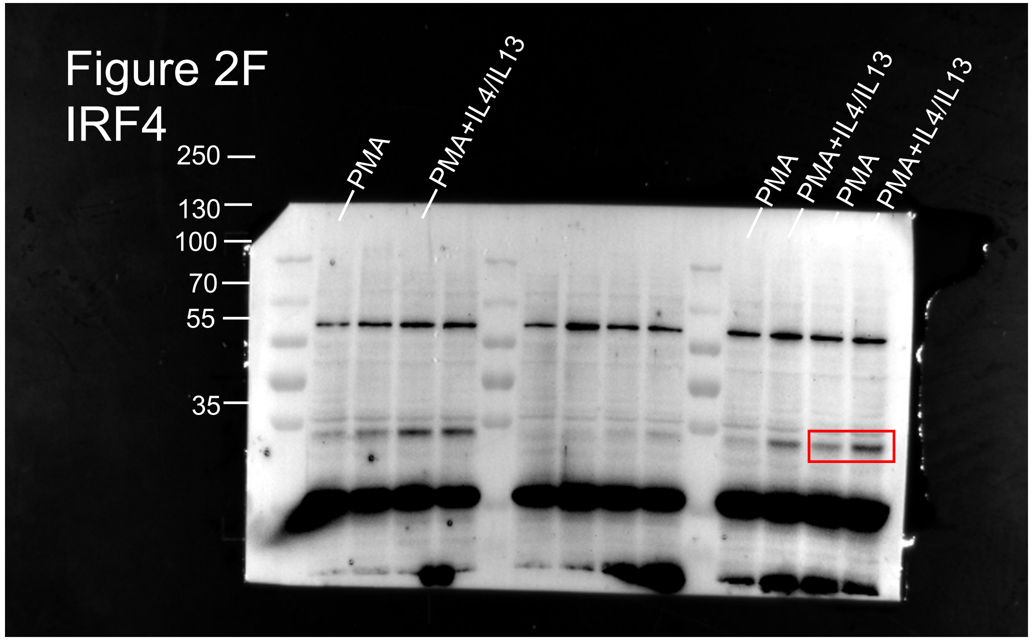
Figure 2F
IRF4
250
130
100
70
55
35
PMA
PMA+IL4/IL13
PMA
PMA+IL4/IL13
PMA
PMA+IL4/IL13

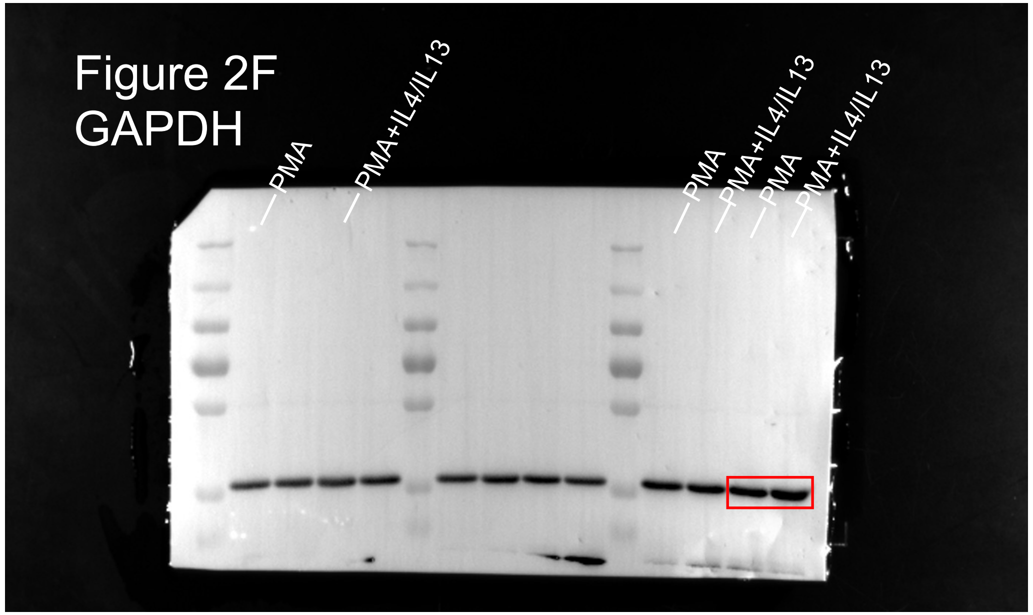
Figure 2F
GAPDH
PMA
PMA+IL4/IL13
PMA
PMA+IL4/IL13
PMA
PMA+IL4/IL13

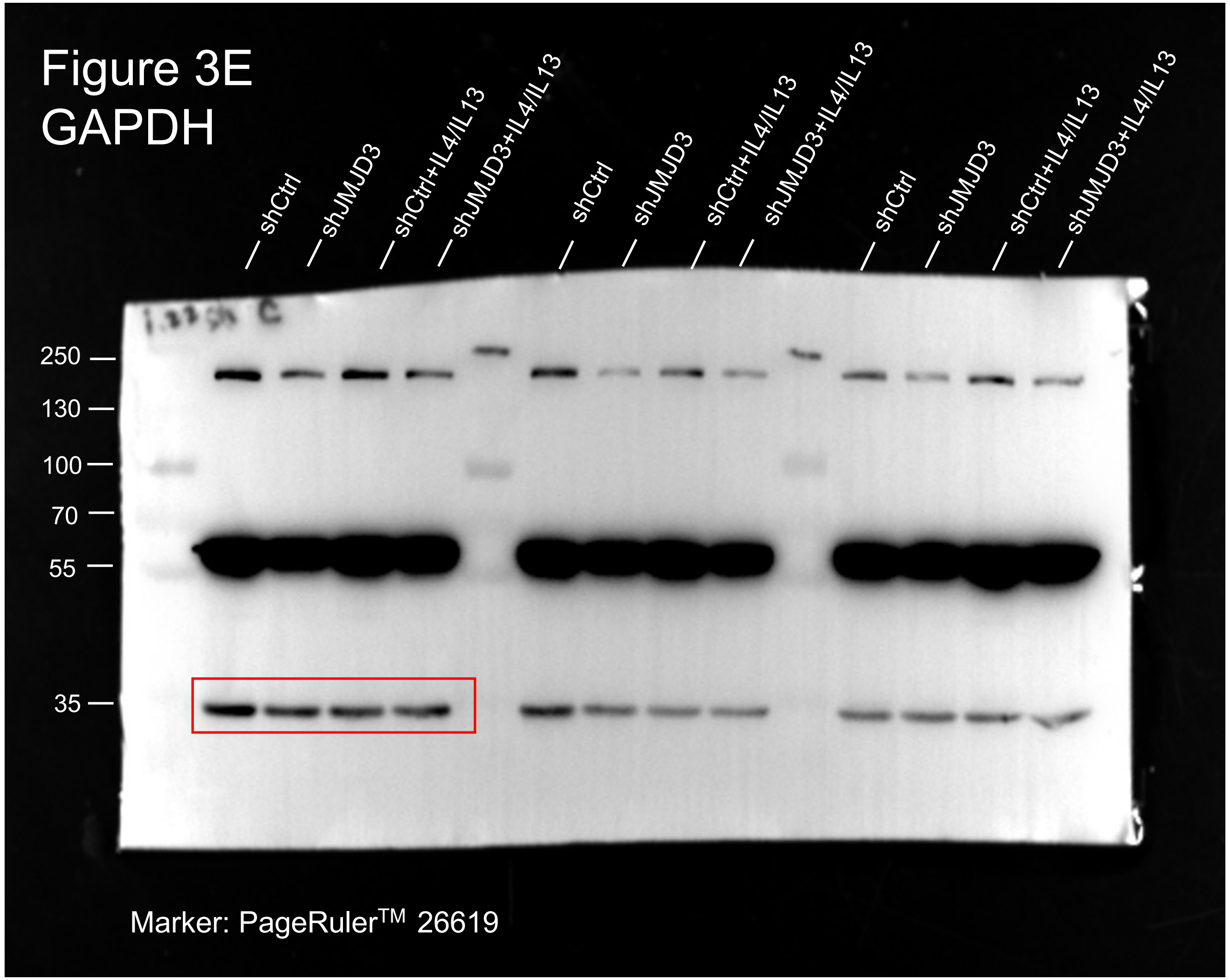
Figure 3E
GAPDH
shCtrl
shJMJD3
shCtrl+IL4/IL13
shJMJD3+IL4/IL13
shCtrl
shJMJD3
shCtrl+IL4/IL13
shJMJD3+IL4/IL13
shCtrl
shJMJD3
shCtrl+IL4/IL13
shJMJD3+IL4/IL13
250
130
100
70
55
35
Marker: PageRuler™ 26619

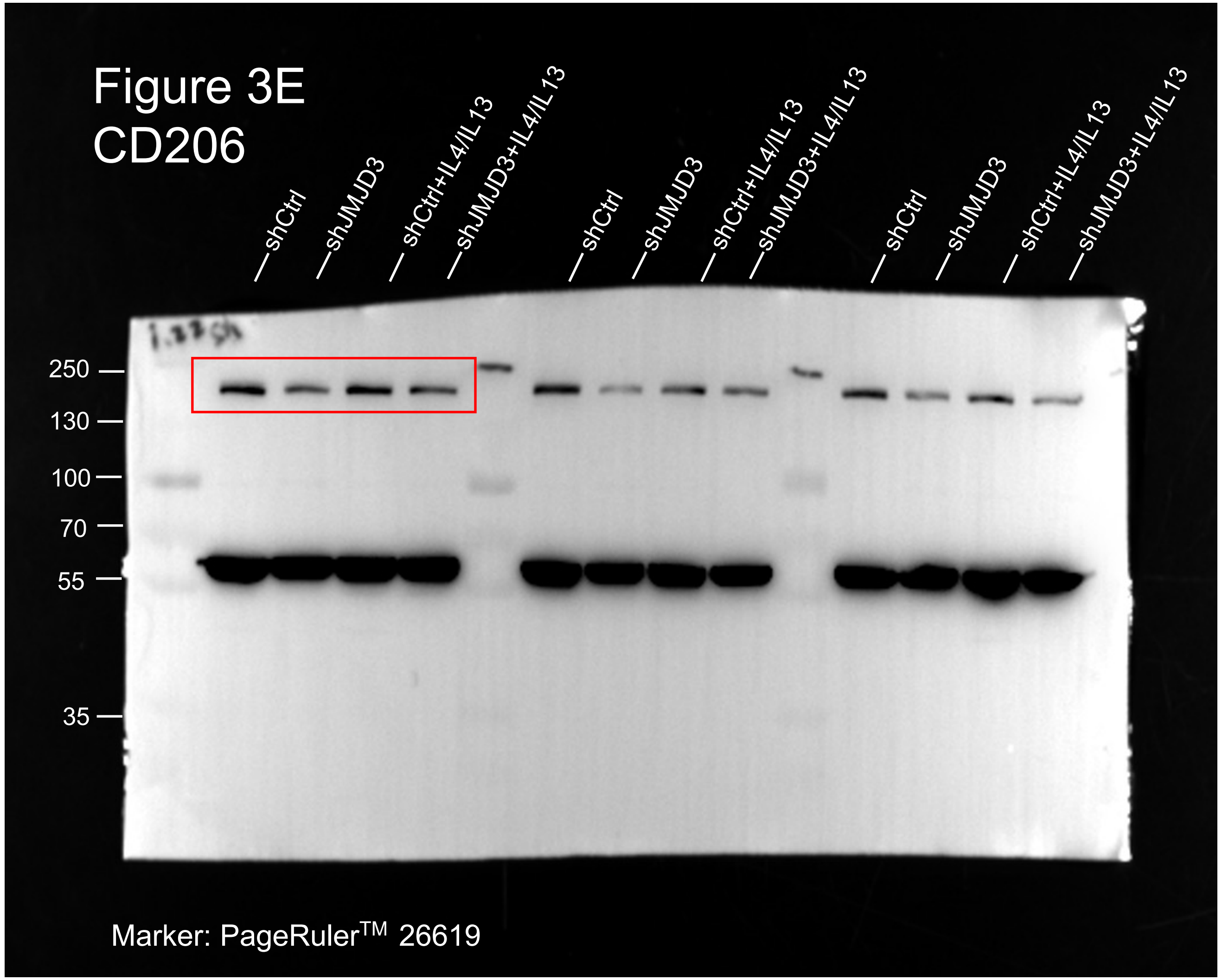
Figure 3E
CD206
shCtrl
shJMJD3
shCtrl+IL4/IL13
shJMJD3+IL4/IL13
shCtrl
shJMJD3
shCtrl+IL4/IL13
shJMJD3+IL4/IL13
shCtrl
shJMJD3
shCtrl+IL4/IL13
shJMJD3+IL4/IL13
250
130
100
70
55
35
Marker: PageRuler™ 26619

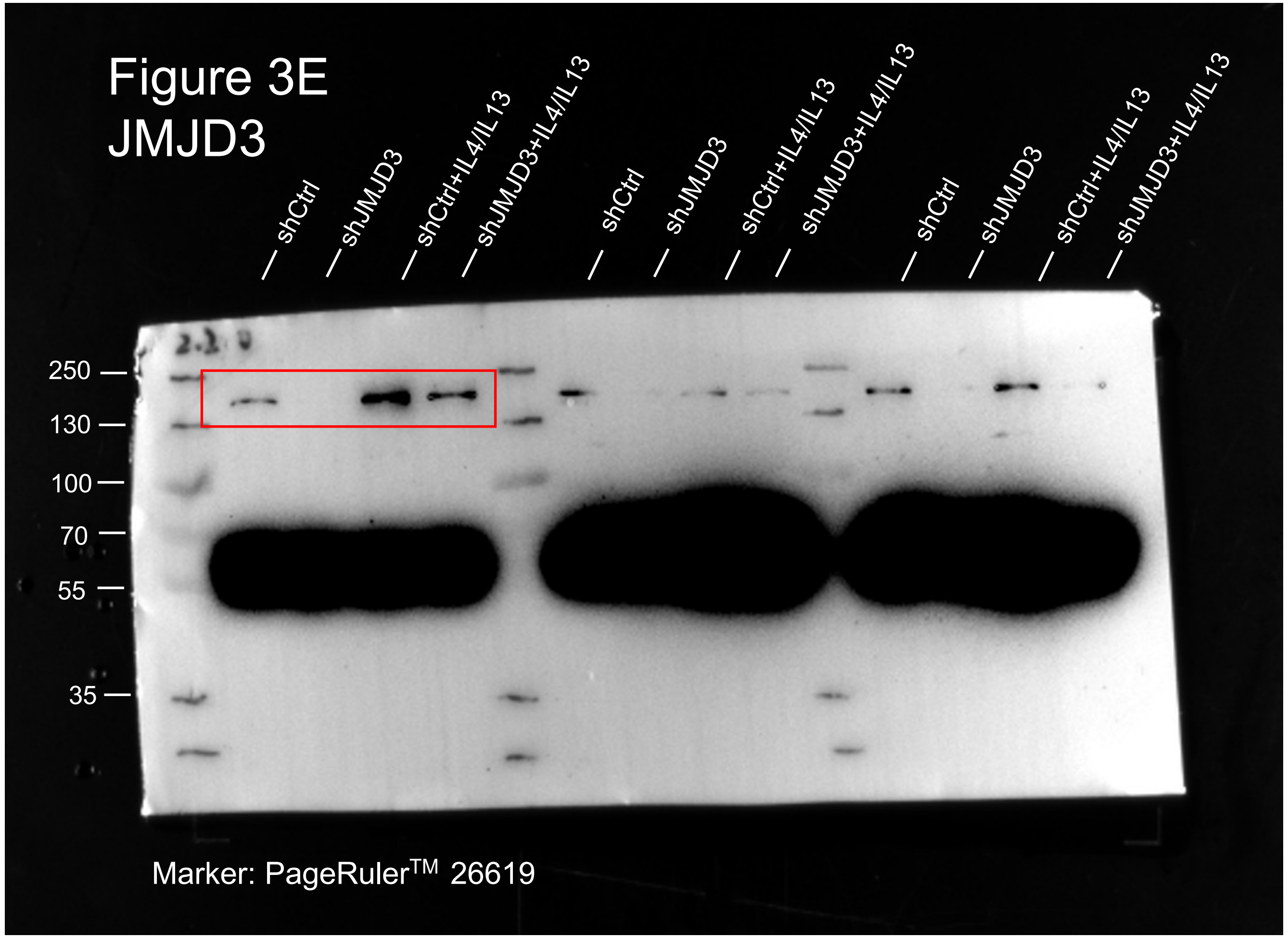

Figure 3E
JMJD3
shCtrl
shJMJD3
shCtrl+IL4/IL13
shJMJD3+IL4/IL13
shCtrl
shJMJD3
shCtrl+IL4/IL13
shJMJD3+IL4/IL13
shCtrl
shJMJD3
shCtrl+IL4/IL13
shJMJD3+IL4/IL13
250
130
100
70
55
35
Marker: PageRuler™ 26619

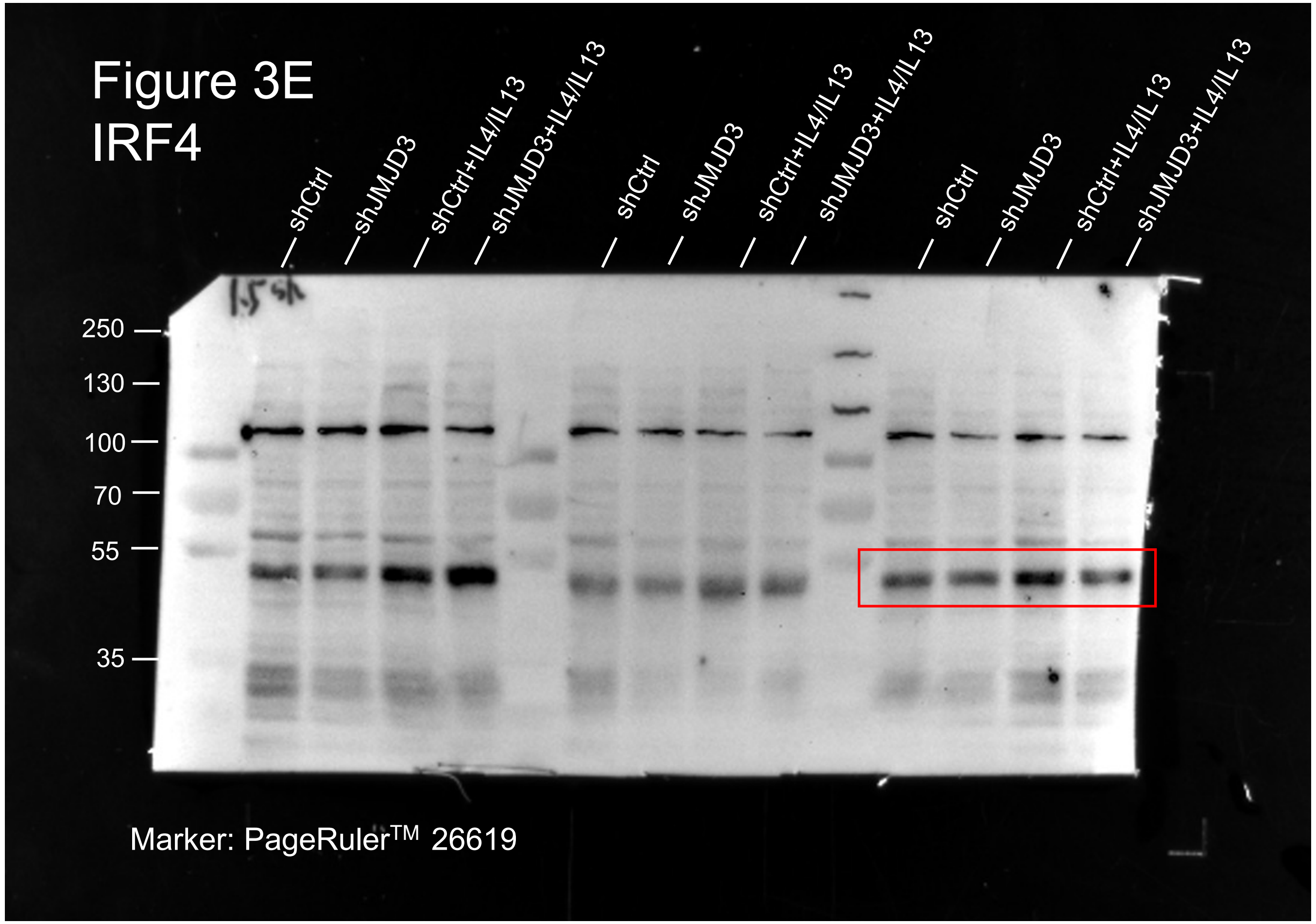
Figure 3E
IRF4
shCtrl
shJMJD3
shCtrl+IL4/IL13
shJMJD3+IL4/IL13
shCtrl
shJMJD3
shCtrl+IL4/IL13
shJMJD3+IL4/IL13
shCtrl
shJMJD3
shCtrl+IL4/IL13
shJMJD3+IL4/IL13
250
130
100
70
55
35
Marker: PageRuler™ 26619

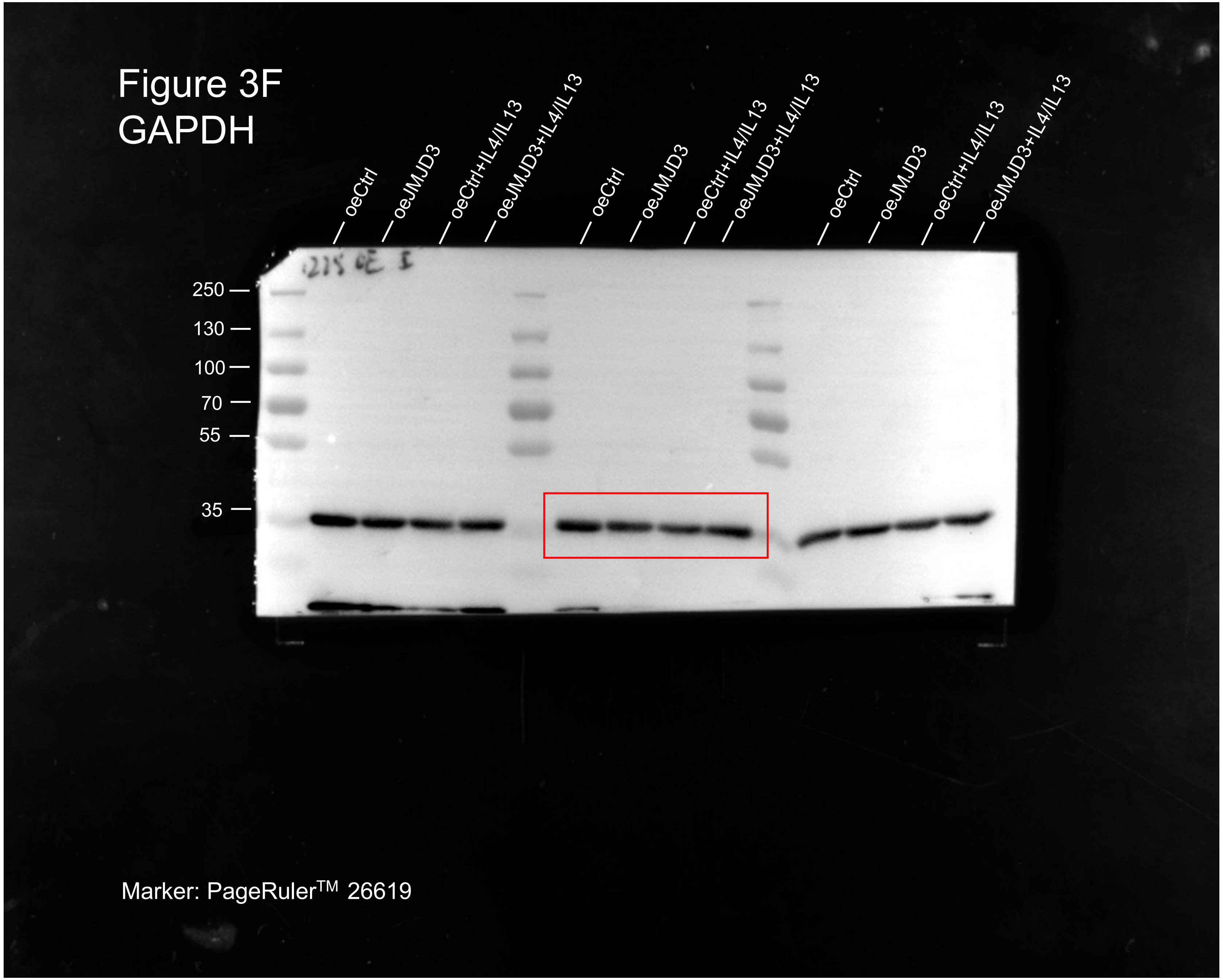
Figure 3F
GAPDH
oeCtrl
oeJMJD3
oeCtrl+IL4/IL13
oeJMJD3+IL4/IL13
oeCtrl
oeJMJD3
oeCtrl+IL4/IL13
oeJMJD3+IL4/IL13
oeCtrl
oeJMJD3
oeCtrl+IL4/IL13
oeJMJD3+IL4/IL13
250
130
100
70
55
35
Marker: PageRuler™ 26619

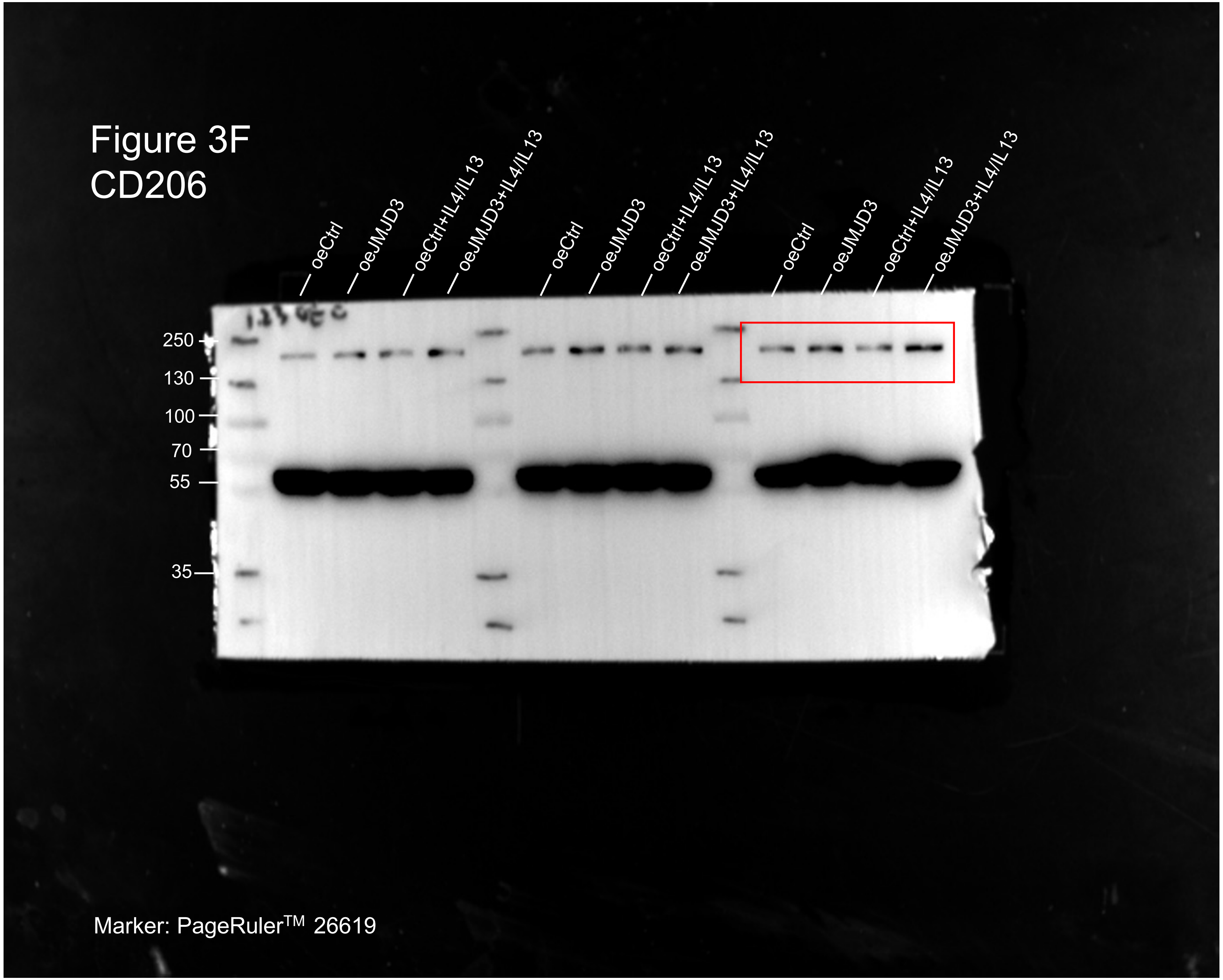

Figure 3F
CD206
oeCtrl
oeJMJD3
oeCtrl+IL4/IL13
oeJMJD3+IL4/IL13
oeCtrl
oeJMJD3
oeCtrl+IL4/IL13
oeJMJD3+IL4/IL13
oeCtrl
oeJMJD3
oeCtrl+IL4/IL13
oeJMJD3+IL4/IL13
250
130
100
70
55
35
Marker: PageRuler™ 26619

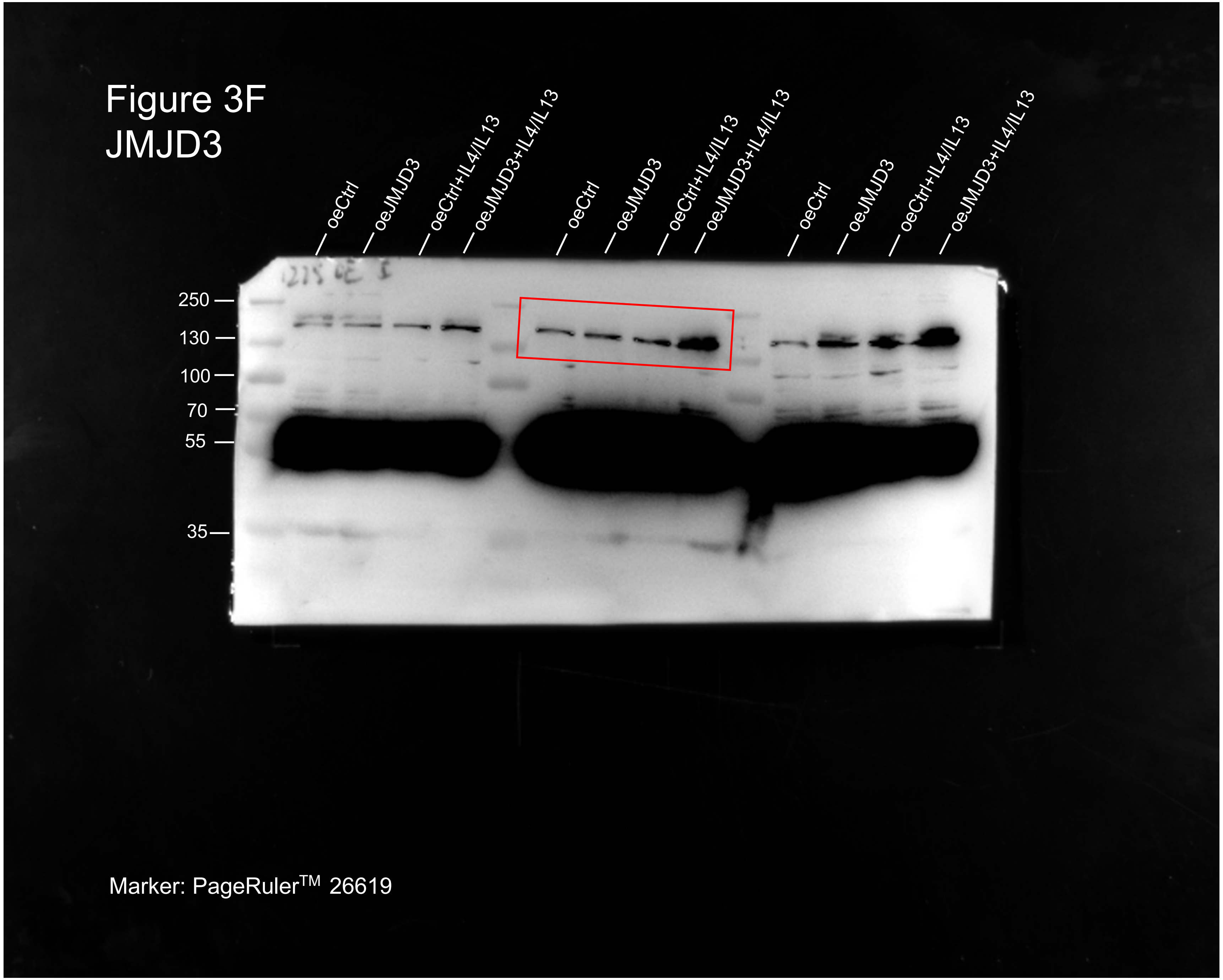
Figure 3F
JMJD3
oeCtrl
oeJMJD3
oeCtrl+IL4/IL13
oeJMJD3+IL4/IL13
oeCtrl
oeJMJD3
oeCtrl+IL4/IL13
oeJMJD3+IL4/IL13
oeCtrl
oeJMJD3
oeCtrl+IL4/IL13
oeJMJD3+IL4/IL13
250
130
100
70
55
35
Marker: PageRuler™ 26619

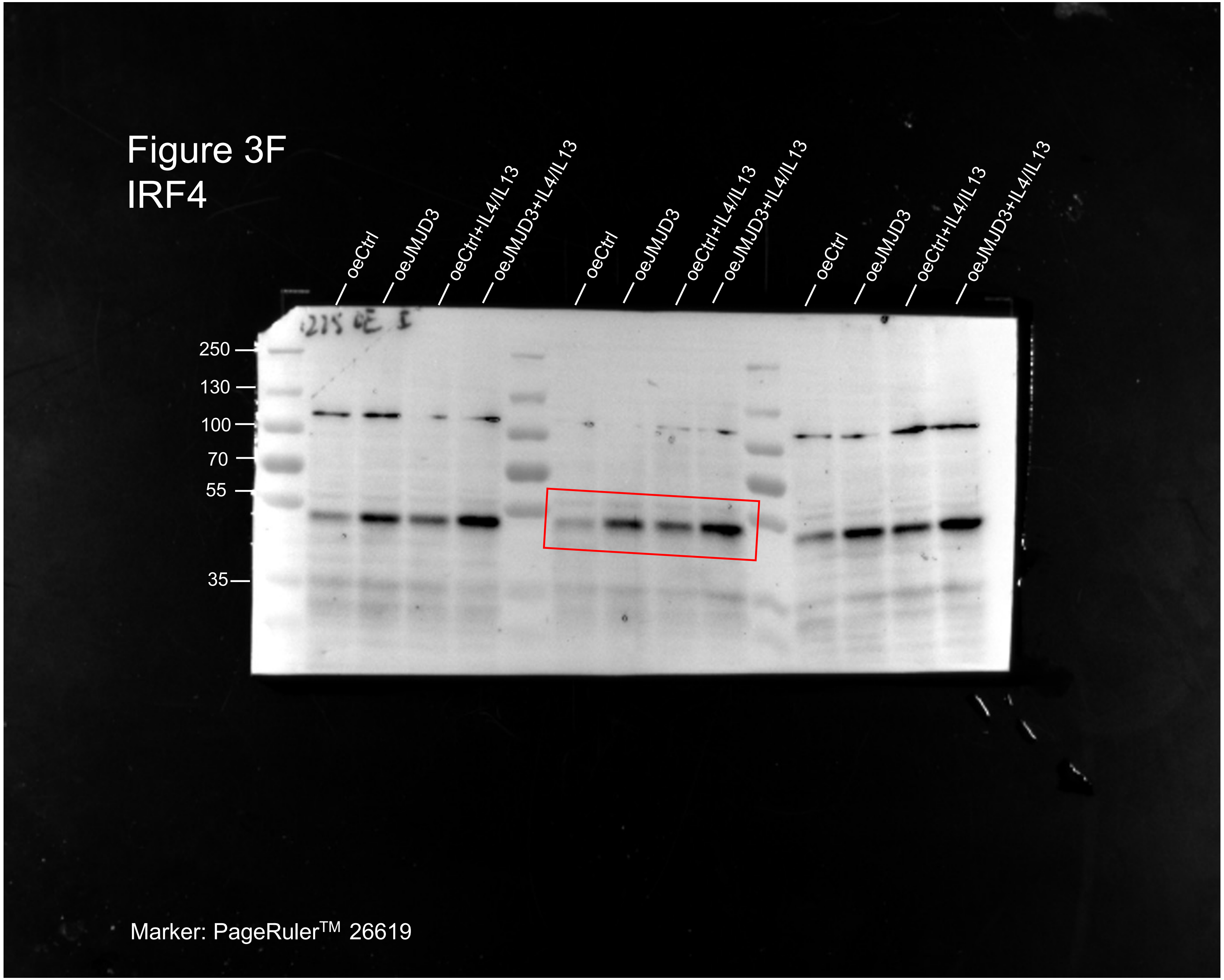
Figure 3F
IRF4
oeCtrl
oeJMJD3
oeCtrl+IL4/IL13
oeJMJD3+IL4/IL13
oeCtrl
oeJMJD3
oeCtrl+IL4/IL13
oeJMJD3+IL4/IL13
oeCtrl
oeJMJD3
oeCtrl+IL4/IL13
oeJMJD3+IL4/IL13
250
130
100
70
55
35
Marker: PageRulerTM 26619

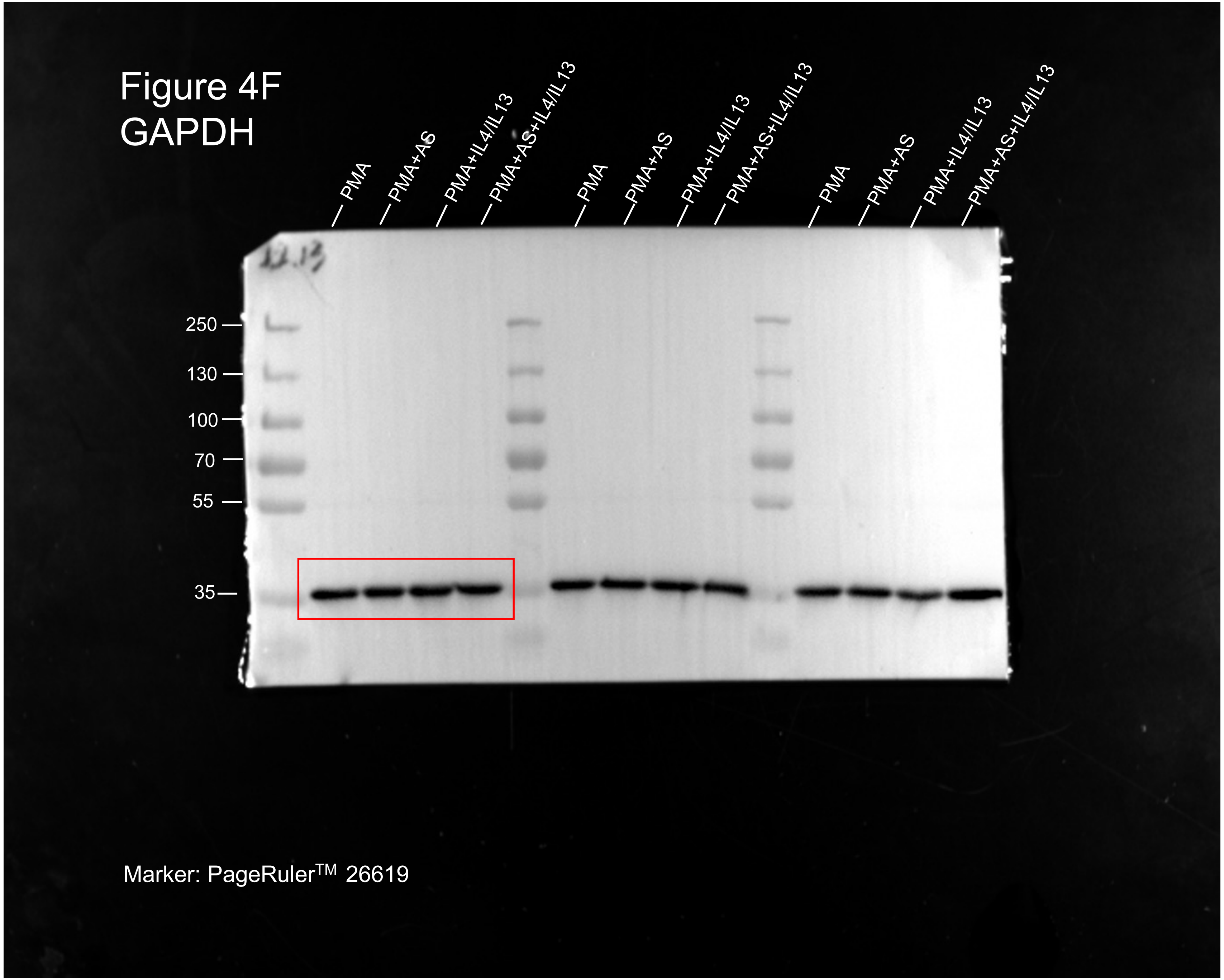
Figure 4F
GAPDH
PMA
PMA+AS
PMA+IL4/IL13
PMA+AS+IL4/IL13
PMA
PMA+AS
PMA+IL4/IL13
PMA+AS+IL4/IL13
PMA
PMA+AS
PMA+IL4/IL13
PMA+AS+IL4/IL13
250
130
100
70
55
35
Marker: PageRuler™ 26619

Figure 4F
JMJD3

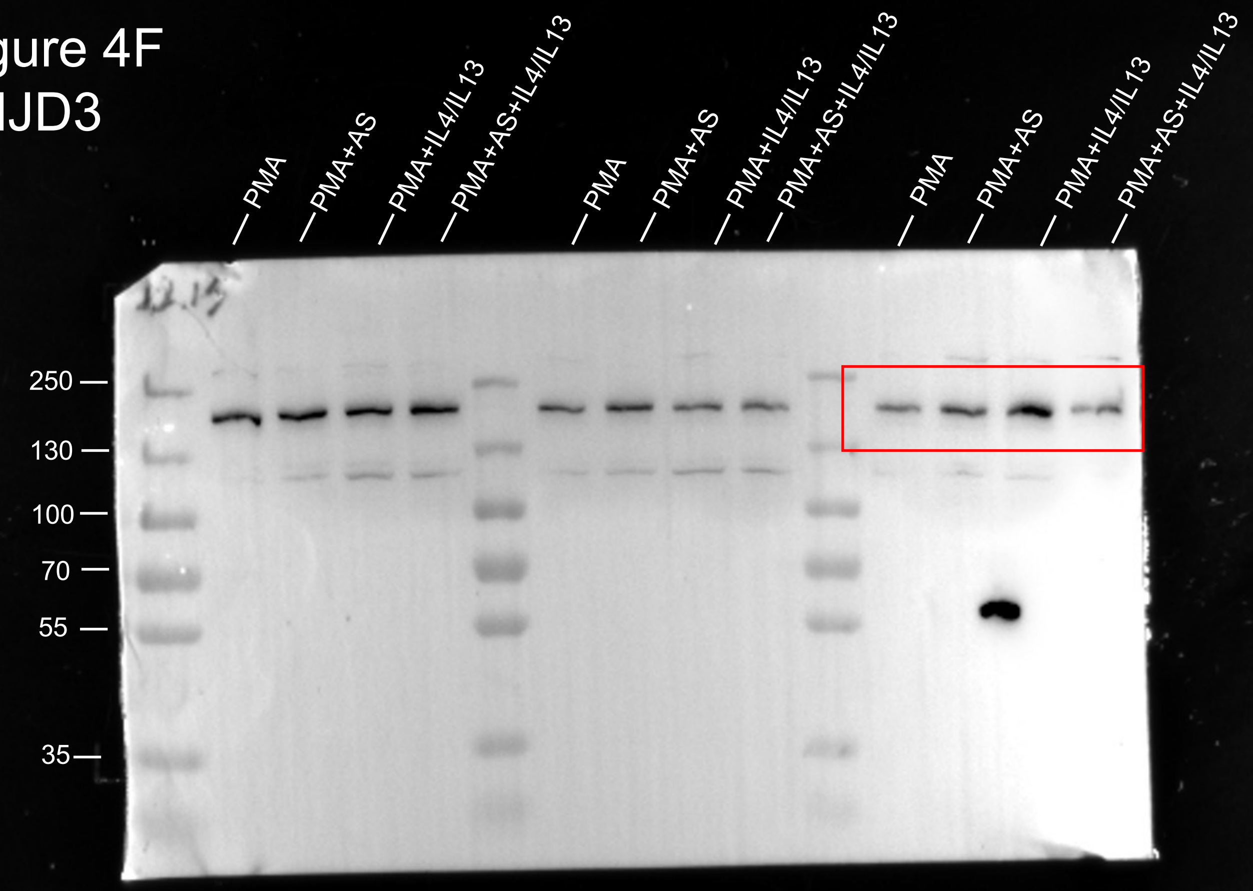

Marker: PageRuler™ 26619

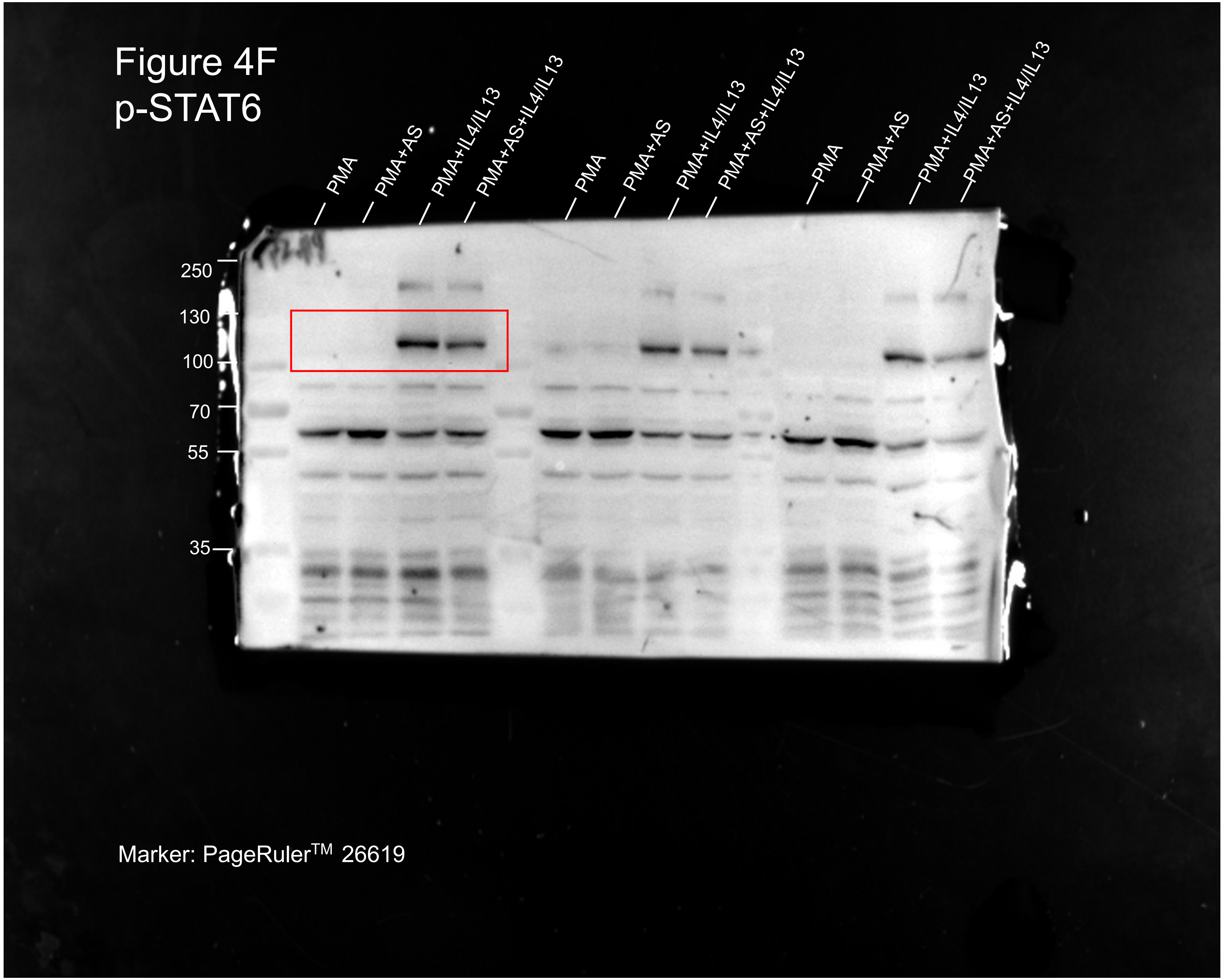
Figure 4F
p-STAT6
PMA
PMA+AS
PMA+IL4/IL13
PMA+AS+IL4/IL13
PMA
PMA+AS
PMA+IL4/IL13
PMA+AS+IL4/IL13
PMA
PMA+AS
PMA+IL4/IL13
PMA+AS+IL4/IL13
250
130
100
70
55
35
Marker: PageRuler™ 26619

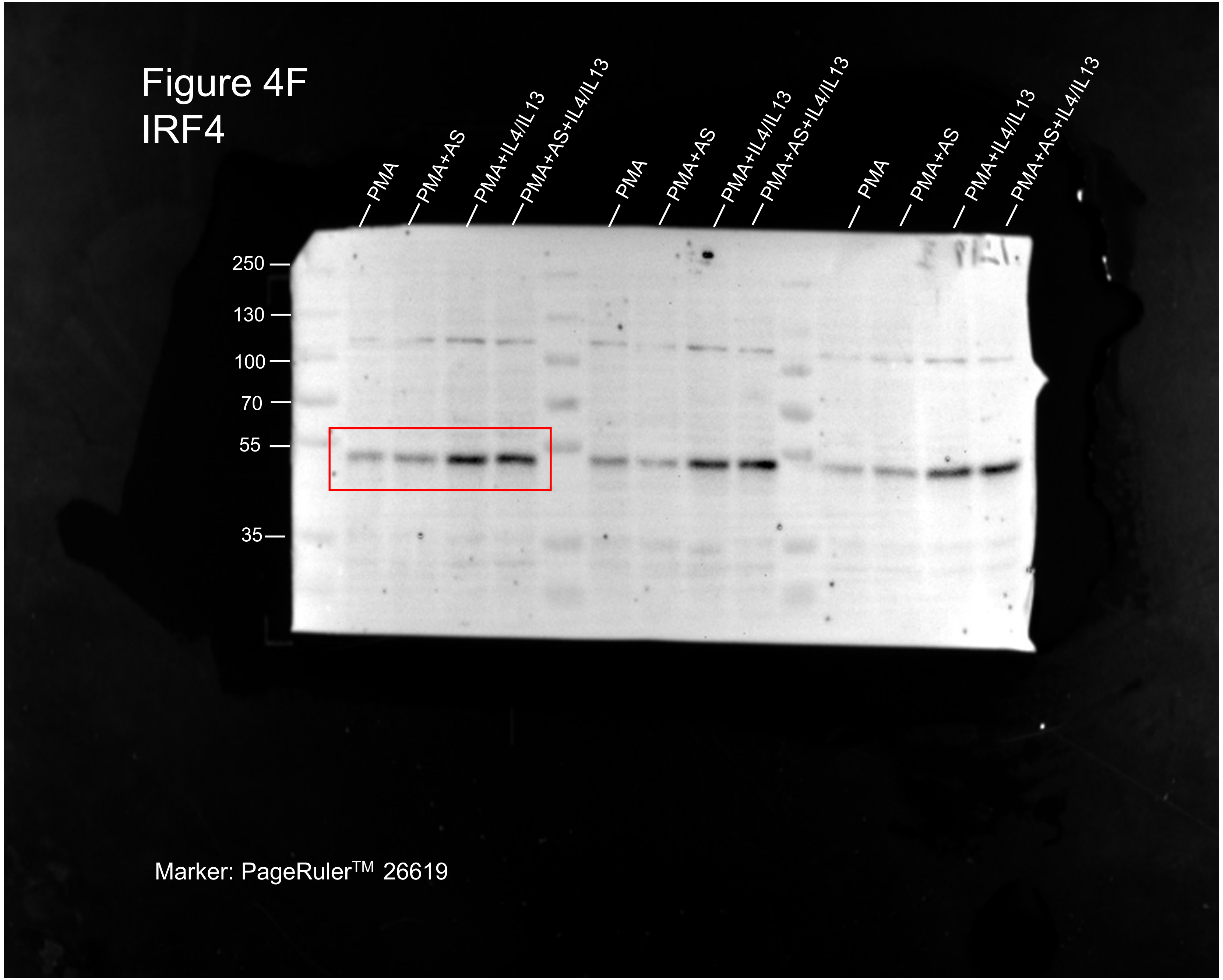
Figure 4F
IRF4
PMA
PMA+AS
PMA+IL4/IL13
PMA+AS+IL4/IL13
PMA
PMA+AS
PMA+IL4/IL13
PMA+AS+IL4/IL13
PMA
PMA+AS
PMA+IL4/IL13
PMA+AS+IL4/IL13
250
130
100
70
55
35
Marker: PageRuler™ 26619

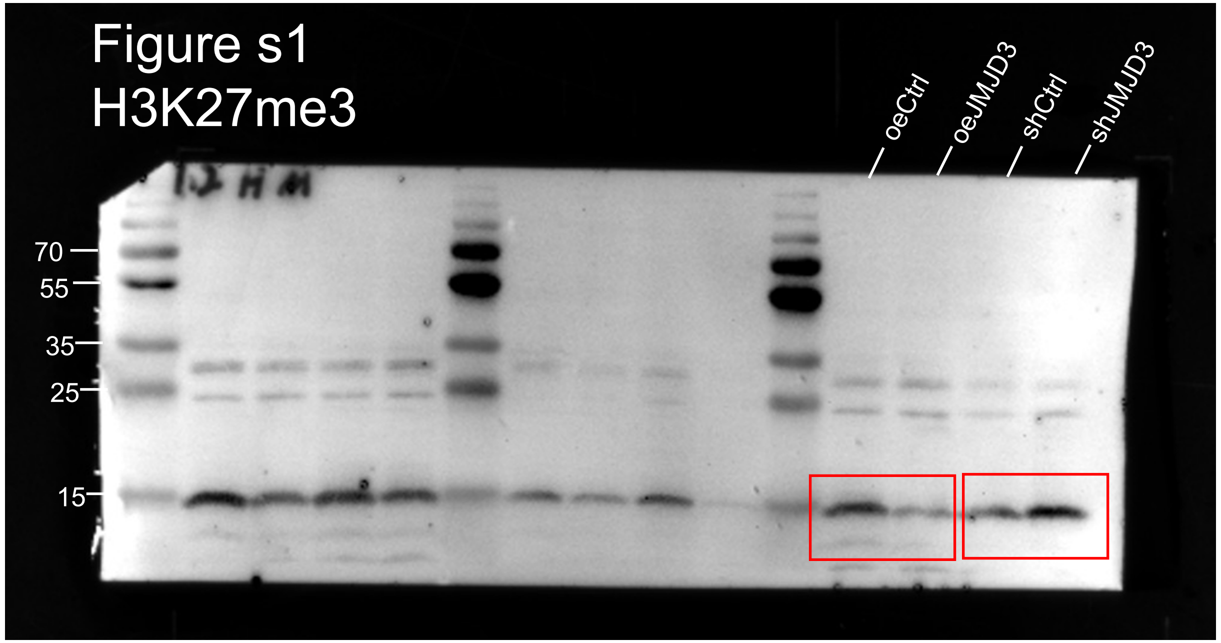
Figure s1
H3K27me3
oeCtrl
oeJMJD3
shCtrl
shJMJD3
70
55
35
25
15

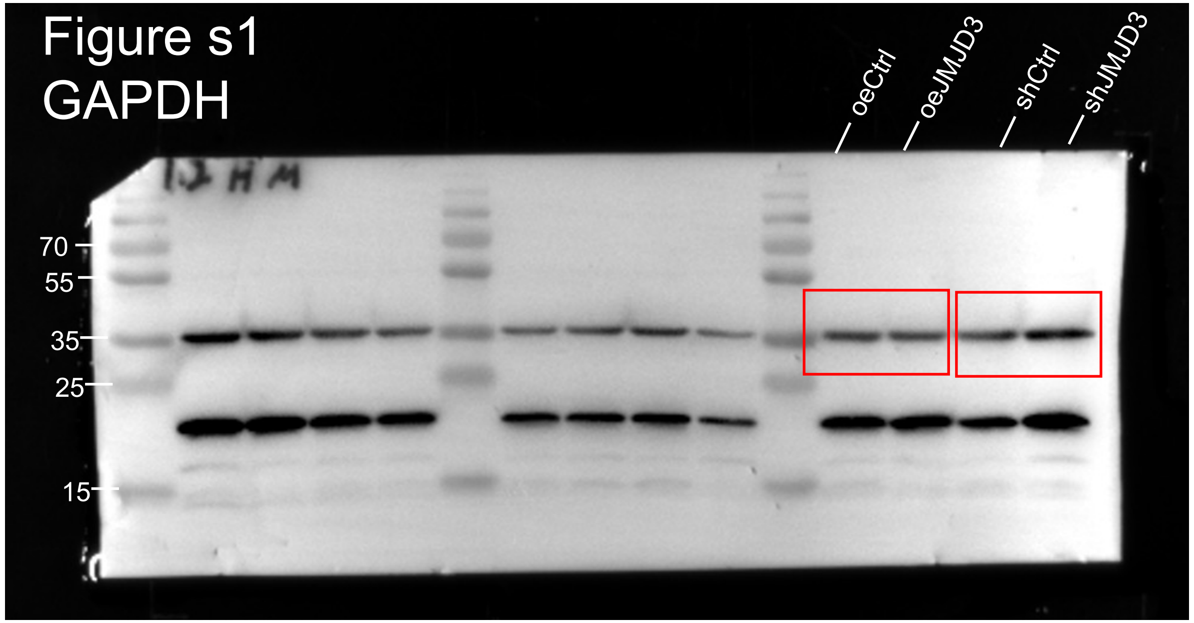
Figure s1
GAPDH
oeCtrl
oeJMJD3
shCtrl
shJMJD3
70
55
35
25
15
