# Supplementary material for: JMJD3 regulates the M2-like macrophage polarization and promotes the growth of breast cancer cells via STAT6/IRF4 axis
Source: PLoS One. 2026 Apr 9;21(4):e0341313. doi: 10.1371/journal.pone.0341313 (PMC13065056; doi:10.1371/journal.pone.0341313)
Supplement: Attachment — Submitted filename: PONE-D-24-45718 - Review suggestions.docx [file pone.0341313.s005.docx]

Review suggestions for “**JMJD3 regulates the M2 macrophage polarization**

**and promotes the growth of breast cancer cells via STAT6/IRF4 axis**”

The authors presented a study to elucidate the molecular mechanism by which JMJD3 protein regulates M2 macrophage differentiation and promotes breast cancer cell growth. The authors identified the role of activated STAT6 in promoting JMJD3 expression and stimulating JRF4 expression to fully differentiate M2 macrophages. The study showed that M2 macrophages have the ability to promote proliferation and inhibit apoptosis in the MDA-MB-231 breast cancer cell line, and that inhibition of JMJD3 or STAT6 significantly reduced M2 macrophage activity. Although the results are promising, there are still some issues that need to be improved or strengthened by further experiments.

1. TCGA analysis showed higher M2 scores in ER-positive and PR-positive patients. However, the functional assay was performed on the MDA-MB-231 cell line (ER-negative, PR-negative). Would the authors could explain about this? Does the STAT6/JMJD3/IRF4 signaling axis have a different or more prominent role in different breast cancer subtypes?
2. The authors should supplement with other cell lines, for example using conditioned media on breast cancer cell lines representing different subtypes such as MCF-7 (ER-positive, PR-positive) or SKBR3 (HER2-positive) to test whether the growth-promoting effect depends on the breast cancer subtype.
3. The study used the pharmacological inhibitor AS1517499 to inactivate STAT6. Small molecule inhibitors can sometimes have off-target effects. To increase the robustness of the results, did the authors consider confirming the results using genetic methods, such as using shRNA or siRNA to knockdown STAT6?
4. The signaling pathway is presented in a linear direction: STAT6 → JMJD3 → IRF4. However, do the authors think it is possible that IRF4 or one of its target genes could negatively regulate the activity of STAT6 or JMJD3?
5. The study demonstrated that JMJD3 knockdown reduced M2 markers. However, the spectrum of macrophage differentiation is considered to be a continuum rather than a discrete M1/M2 state. The question is: does JMJD3 inhibition simply block the M2 differentiation pathway, or does it actively push macrophages towards M1 polarization?
6. The authors could examine M1-specific markers (e.g., iNOS, IL-12) in JMJD3 knockdown cells, as this would provide further insight into the role of JMJD3 as a “switch” that directs polarization.
7. JMJD3 is a histone demethylase that can act on many different genes, not just IRF4. The study focused on the JMJD3/IRF4 axis. So do the authors think that JMJD3 can regulate other genes involved in M2 macrophage function in parallel with regulating IRF4?
8. The authors suggest that STAT6 inhibition reduces JMJD3 expression, suggesting that STAT6 regulates transcription of the JMJD3 gene. However, this is only an indirect relationship. Experiments are needed to demonstrate whether STAT6 acts on the promoter region of the JMJD3 gene to activate transcription (Chromatin Immunoprecipitation - ChIP).
9. The study only used conditioned medium to demonstrate that M2 macrophages affect cancer cells through the soluble factors they secrete. However, direct physical interactions between the two cell types may also play an important role. Direct co-culture of macrophages with breast cancer cells could be used to assess whether the observed effects are enhanced.
10. The authors have not specified which factors secreted by M2 are primarily responsible for promoting cancer cell growth.
